# Functional classification of protein toxins as a basis for bioinformatic screening

**DOI:** 10.1038/s41598-017-13957-1

**Published:** 2017-10-24

**Authors:** Surendra S. Negi, Catherine H. Schein, Gregory S. Ladics, Henry Mirsky, Peter Chang, Jean-Baptiste Rascle, John Kough, Lieven Sterck, Sabitha Papineni, Joseph M. Jez, Lucilia Pereira Mouriès, Werner Braun

**Affiliations:** 1 Sealy Center for Structural Biology and Molecular Biophysics, Department of Biochemistry and Molecular Biology, University of Texas, Medical Branch, Galveston, TX 77555-0304 USA; 2 Foundation for Applied Molecular Evolution, Inc., Alachua, FL 32615-9495 USA; 3 DuPont Haskell Laboratory, 1090 Elkton Road, Newark, DE 19711 USA; 4 Pioneer Hi-Bred, DuPont Agricultural Biotechnology, 200 Powder Mill Road, Wilmington, DE 19880 USA; 5 Bayer SAS, 355 rue Dostoïevski, CS 90153, Valbonne, 06906 Sophia Antipolis France; 6 Office of Pesticide Programs, Microbial Pesticides Branch, US Environmental Protection Agency, Washington, DC USA; 7 Department of Plant Systems Biology, Department of Plant Biotechnology and Bioinformatics, Ghent University, B–9052 Ghent, Belgium; 8 Dow AgroSciences LLC, 9330 Zionsville Road, Indianapolis, IN 46268 USA; 9 Department of Biology, Washington University in St. Louis, One Brookings Drive, CB 1137 St. Louis, MO USA; 10 ILSI Health and Environmental Sciences Institute (HESI), 1156 Fifteenth St., NW, Washington, DC 20005 USA

## Abstract

Proteins are fundamental to life and exhibit a wide diversity of activities, some of which are toxic. Therefore, assessing whether a specific protein is safe for consumption in foods and feeds is critical. Simple BLAST searches may reveal homology to a known toxin, when in fact the protein may pose no real danger. Another challenge to answer this question is the lack of curated databases with a representative set of experimentally validated toxins. Here we have systematically analyzed over 10,000 manually curated toxin sequences using sequence clustering, network analysis, and protein domain classification. We also developed a functional sequence signature method to distinguish toxic from non-toxic proteins. The current database, combined with motif analysis, can be used by researchers and regulators in a hazard screening capacity to assess the potential of a protein to be toxic at early stages of development. Identifying key signatures of toxicity can also aid in redesigning proteins, so as to maintain their desirable functions while reducing the risk of potential health hazards.

## Introduction

Most genetically engineered (GE) food crops involve expressing an introduced protein, thus assessing the safety of the protein is required before commercialization^1–4^. GE crops are created by introducing gene(s) from one species into a crop plant species to improve the nutritional value, yield, drought resistance, herbicide tolerance or pest resistance. Biotechnology companies screen new constructs early in the product development process in order to remove potential hazards and ensure the safety of their product pipelines. National and international regulatory agencies have established guidelines for assessing both trait and GE crop safety through a weight of evidence approach^5–7^. The US-FDA, EPA, USDA, or EFSA and other international organizations require scientifically validated methods to ensure reliable results are generated that allow them to assess the safety of introduced proteins in GE crops.

*In silico* methods and webservers have been successfully developed to predict toxicity of small molecular weight compounds. These include the systems pharmacology approach^8^ to predict drug toxicity and the EPA ToxCast program^9,10^ to screen chemicals for potential toxicity to human and the environment. Similar broadly offered bioinformatics tools are not available to predict whether a protein poses the potential to have a toxic effect on mammals. One of the reasons is the absence of a comprehensive, publicly available database containing all proteins with experimentally verified toxic effects in humans or animal studies. Specific databases exist for animal toxins^11,12^, spider venoms^13^ and microbial pathogens^14^. Although the amino acid sequence determines the three-dimensional structure and the biochemical function of the protein, the specific determinants for the pathogenic effect are not known in many cases. Further, the amino acids that dictate toxic function may be quite distant from one another in the linear sequence of the protein, but may be close together within the folded three-dimensional structures of the proteins. These residues may be also distributed on several protein domains, or even on different monomers in multidomain protein toxins.

The extensive data sets of amino acid sequences, three-dimensional structures, biochemical and biological functions of gene products in publicly available databases can be the basis for bioinformatics approaches to determine the potential risk of toxicity. Proper cataloguing of this data, by discriminating the small proportion of proteins that are known toxins, is one part of an overall “weight-of-evidence” evaluation for the safety of GE products^4,5,15,16^. Here we document the first steps to establish a bioinformatics strategy for evaluating the toxic potential of a protein. Beginning with a manually curated list obtained through a keyword search, over 10,000 protein sequences were grouped based on their sequence identity, and then according to their similarity to protein families as classified in the PFAM database^17^. The clustering was automatically performed by a series of independent single linkage clustering with varying thresholds for sequence identities and the top 100 clusters manually inspected for common biochemical and physiological functions. In addition, for all toxin entries protein domains were assigned to PFAM classes. Both procedures indicated that there are only a limited number (< 400) of potential mechanisms for protein toxicity. The current list is a starting point for a relational database of protein toxins for hazard screening. We show further that sequence alignments of the clustered toxins can establish structural and sequential motifs^18–20^ for use in distinguishing toxins from their non-toxic homologues in the same PFAM class. Extending this classification and motif analysis to all known toxic proteins can aid in identifying possible mechanisms of toxicity during the first tier of hazard screening, and prevent potentially problematic proteins from entering the developmental pipeline.

## Results

### Selection of a representative set of toxin sequences

**Figure 1:**
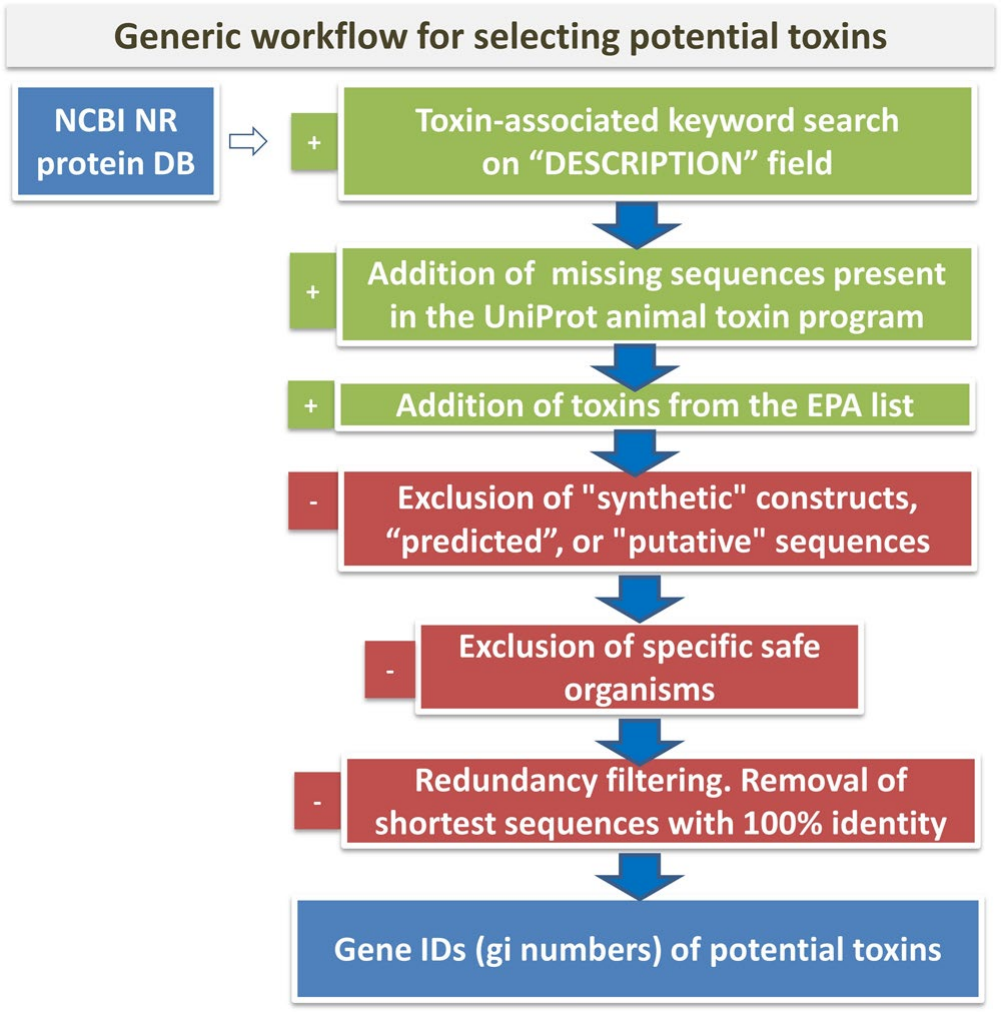
Workflow for selecting potential toxin sequences included in the database. Different selection of keywords were combined to provide a broad coverage of toxins.

We began with a curated list of proteins whose signatures contained one of a selected series of key words that indicated protein toxicity (Fig. 1). The list was simplified by removing duplicates, putative or synthetic constructs, and by adding missing sequences catalogued in existing toxin databases, such as that maintained by the EPA (see materials and methods). The final list of the toxins contained 10,389 protein sequences. Sequence clustering and analysis is described in the Methods section.

### About 400 clusters at the 35% sequence identity level contain most protein toxins

To examine the sequence variability of the extracted ~10,000 protein toxins, we generated clusters with cutoff levels at 5% intervals and manually analyzed the most populated individual clusters at the 95%, 65% and 35% sequence identity levels. Clusters at the 95% level were used to identify and remove highly redundant toxin sequences. Sequences with 35% identity can be generally considered to have similar 3D-structures^21–23^. This observation of structural similarity has been confirmed in many cases by the results of Protein Structure Initiative of the NIH^24,25^.

**Figure 2:**
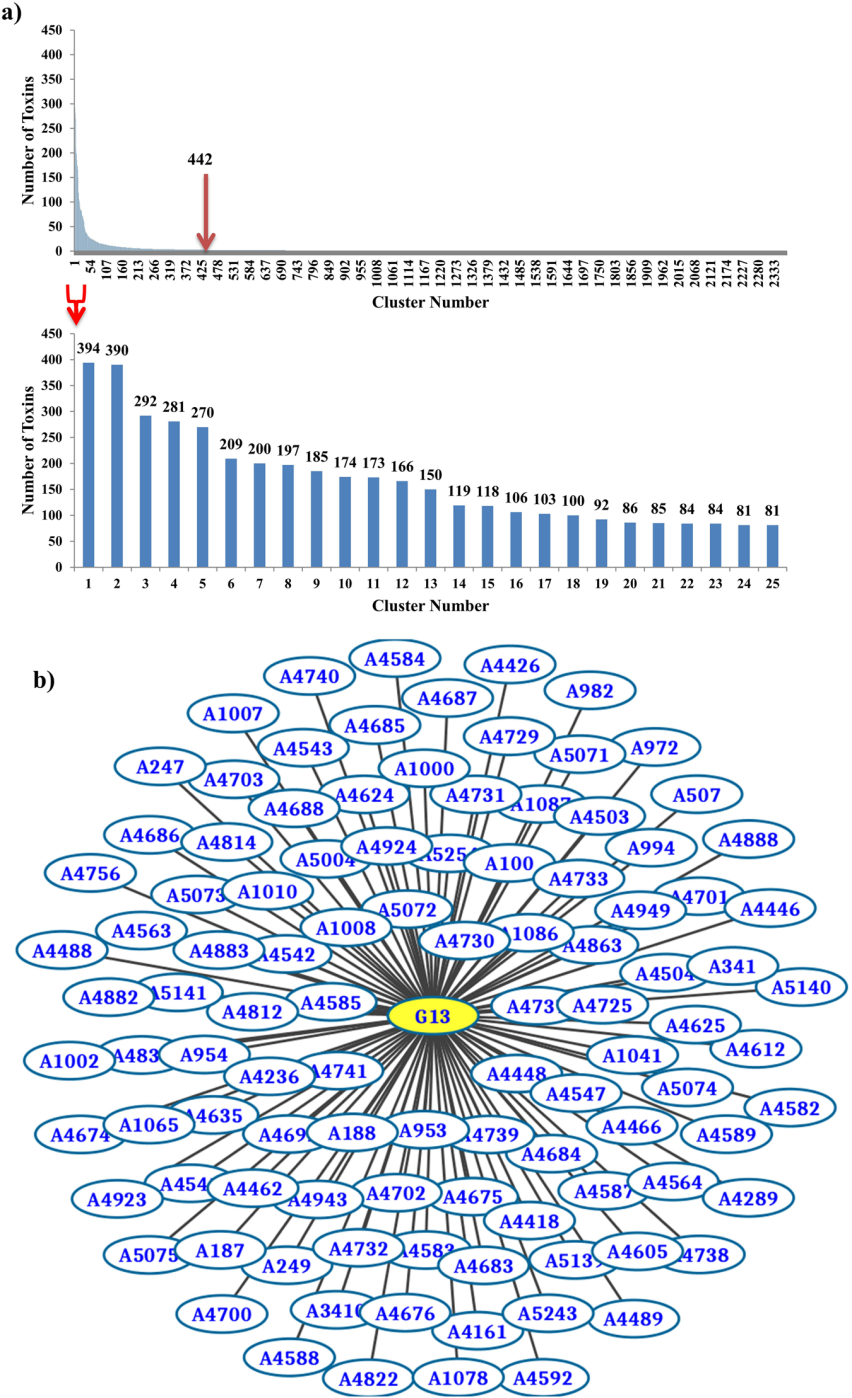
Cluster and network analysis of protein toxin sequences: (**a**) Toxin sequences in each of the 35% sequence identity clusters are shown. Most sequences were contained in about 442 clusters. (**b**) Relation of a cluster at the 95% sequence identity level (indicated by Axxxx) to larger clusters at 35% sequence identity level. The example shows a 35% cluster of conotoxin sequences (G13) composed of multiple 95% clusters.

However, functional similarity cannot be easily deduced from a simple sequence similarity cutoff. Even proteins that are very similar in both sequences and structures may have completely different functions, whereby only one group is toxic. We therefore used here an empirical approach with a varying sequence identity level to learn about the cluster properties of known protein toxins. The number of clusters decreased from a high of 6,295 at 95% to 3,562 at 65% sequence identity, and to 2,375 when grouped at 35% identity. The 335 most populated clusters at the 35% identity level contain about 80% of the protein toxins (Fig. 2a). The rest of the toxins formed very small clusters or were unique sequences at this cut-off level.

### Annotations may distinguish members within a cluster that are functionally related

Not surprisingly, the most closely related proteins in the most populated clusters had similar functions, although the annotations in the NCBI entry data files were sometimes quite different. For example, the highly similar proteins in the second highest populated cluster were called Shiga toxin 2 A or verocytotoxin 2 (Table S1), whereby both terms indicate similar activity. Analysis of the clustering at 65% and 35% identity indicated that although the clusters were progressively larger, they still represented relatively homogeneous groups of proteins. For example all of the 394 entries in the top cluster at 35% identity are conotoxins (Table S2).

### Functional relations of toxins within clusters

Comparison of the most populated clusters of bacterial toxins at the 35% sequence identity showed that our clustering was consistent with functional annotation. We manually analyzed the bacterial toxins in the top 100 clusters at 65% and 35% identity (Table S3). All toxins within the same 35% cluster were functionally related, despite diverse nomenclature used in the NCBI annotations. Proteins with similar annotations that clustered independently at 35% also often had biologically distinct functions. We thus undertook further analysis with PFAM, as discussed below.

### Hierarchical relation between clusters at different sequence identity levels

To illustrate the relationship between the clusters at 95% and those at 65% or 35% sequence identity level, we used a network analysis. Each cluster at different sequence identity threshold was represented by a group ID, and the relationships between these groups were visualized using Cytoscape^26^ (Fig. 2b). Comparison of these networks at different sequence identity levels showed that the larger clusters at 35% represent toxins within the same protein family or protein superfamily. We thus suggest that the 35% sequence identity level represents a good choice for a functional grouping of the toxins, and the 95% levels can be used to resolve nomenclature issues.

### Unique sequences are short or partial sequences

**Figure 3:**
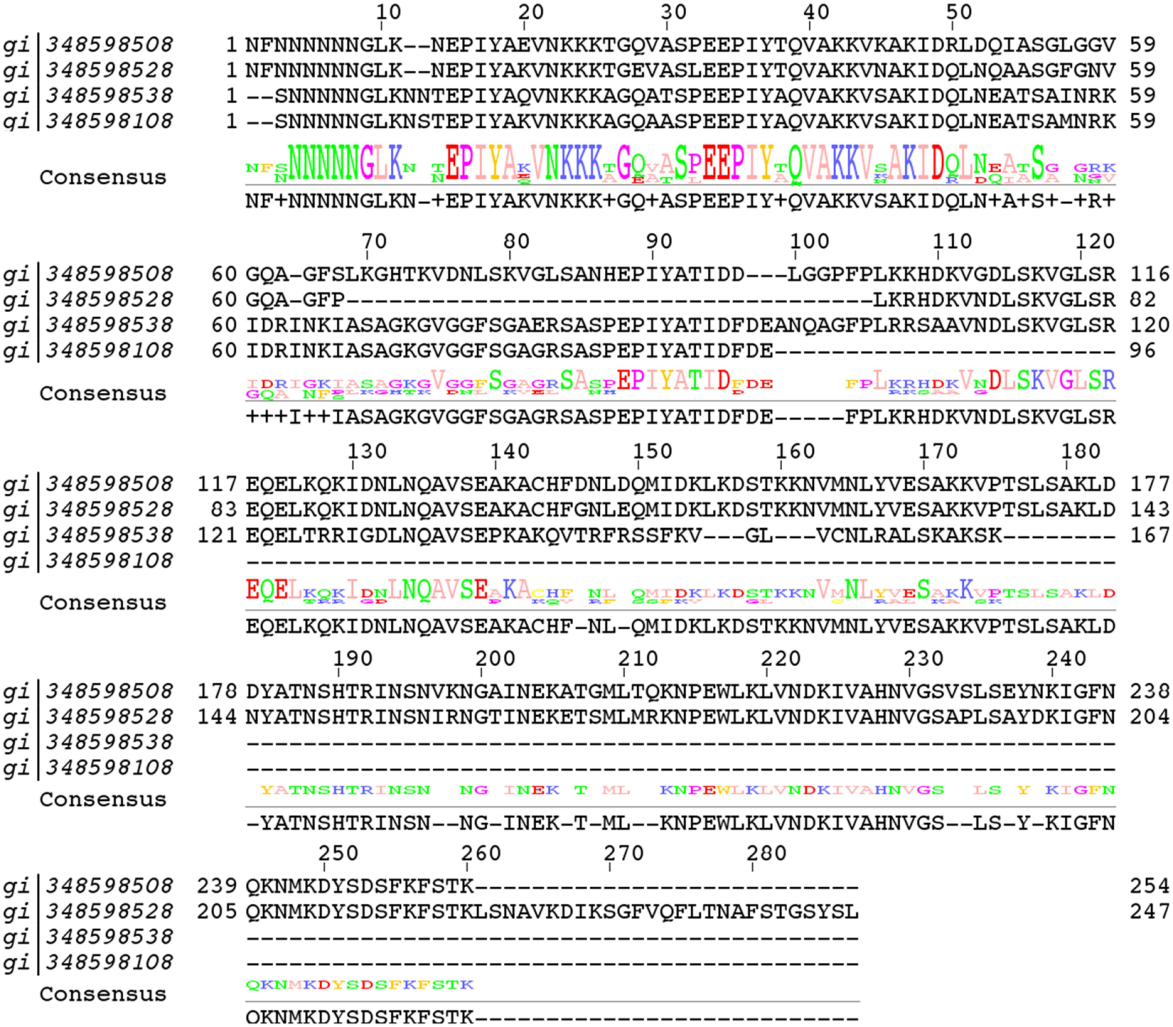
Most of the 1600 singlet sequences can be related to highly populated clusters using multiple sequence alignments. For example, the first sequence is a cytotoxin associated protein from *Heliobacter plyori* included in cluster 4, the other three are from different clusters with only one sequence. Those sequences are almost identical to the first sequence, but contain deletions from 69 to 105, or at the C-termini.

Manual analysis of the clusters with only one sequence indicated that the overwhelming majority of those singlet sequences were fragments of whole toxins that were contained with high sequence identity in a larger cluster. For example, cluster number 4 at the 35% identity level contains 281 cytotoxin-associated proteins. Three singlet sequences could be aligned with the N-terminal of these sequences, but were too short to make the 35% cutoff for identity with the whole, much longer sequences within the cluster (Fig. 3). Thus, we can assign those entries manually to cluster 4.

### The functional diversity of toxins is dramatically restricted

Most bacterial toxins are multidomain proteins, where only one domain may contain the enzymatic region responsible for their detrimental effects. Other toxins form oligomeric assemblies that form membrane pores or need specific protein receptors or lipids to initiate the oligomerization process^27^. Sequence similarity classification alone will thus be misleading for many proteins. Thus the database was further organized according to functional domains, using the PFAM classification^28,29^. Here, toxin identity to discrete areas in the protein known to be responsible for a given function is more important than absolute overall sequence similarity. In addition to expert curation of well-studied proteins, PFAM uses a Hidden Markov Model (HMM) approach, whereby patterns of amino acids in parts of the sequence can reveal similar function. Most of the toxins in the database could be automatically classified by the HMMER software^30^ according to the PFAM classification of their domains.

**Figure 4:**
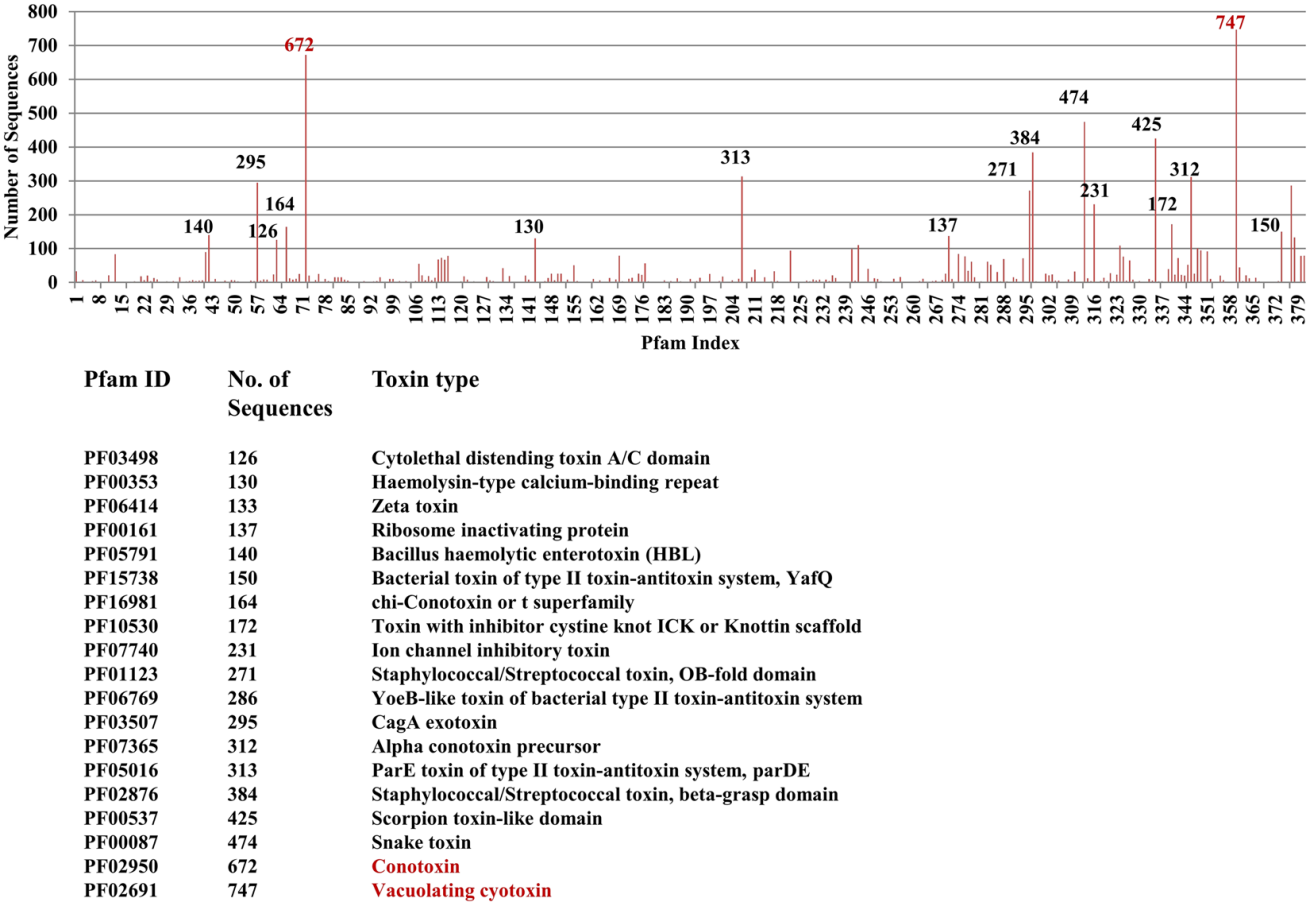
The toxin sequences grouped functionally to 381 PFAM domains. The number of sequences in each PFAM class varied widely (top). The most populated PFAM domains with the number of sequence entries are listed below.

The automated search with HMMER assigned 8570 toxins in the database to only 381 different protein domains out of 16,295 annotated domains in the PFAM database. For the remaining toxins we could manually assign about 500 entries within the same list of protein domains by association, based on their 35 or 65% identity to assigned sequences within the same clusters. This result is also consistent with our cluster analysis at the 35% level, where < 400 clusters accounted for most of the sequences. The functional building blocks of toxins thus come only from a limited subset of domains generally found in proteins. The functional domains found in the protein sequences correspond to less than 3% of the 16,295 annotated domains in the PFAM database. The number of sequences in the database in each PFAM domain is highly variable (Fig. 4). Also consistent with the cluster analysis, the most populated PFAM domains are from vacuolating cytotoxins, conotoxins, snake, scorpion and bacterial toxins. This list of PFAM domains and the associated HMM profiles could be a starting point to find more related toxins in public databases when used with selected keywords. A complete list of all 381 PFAM domains is given as supplementary material (Table S4).

### Domain structures of toxins are critical for their function

**Figure 5:**
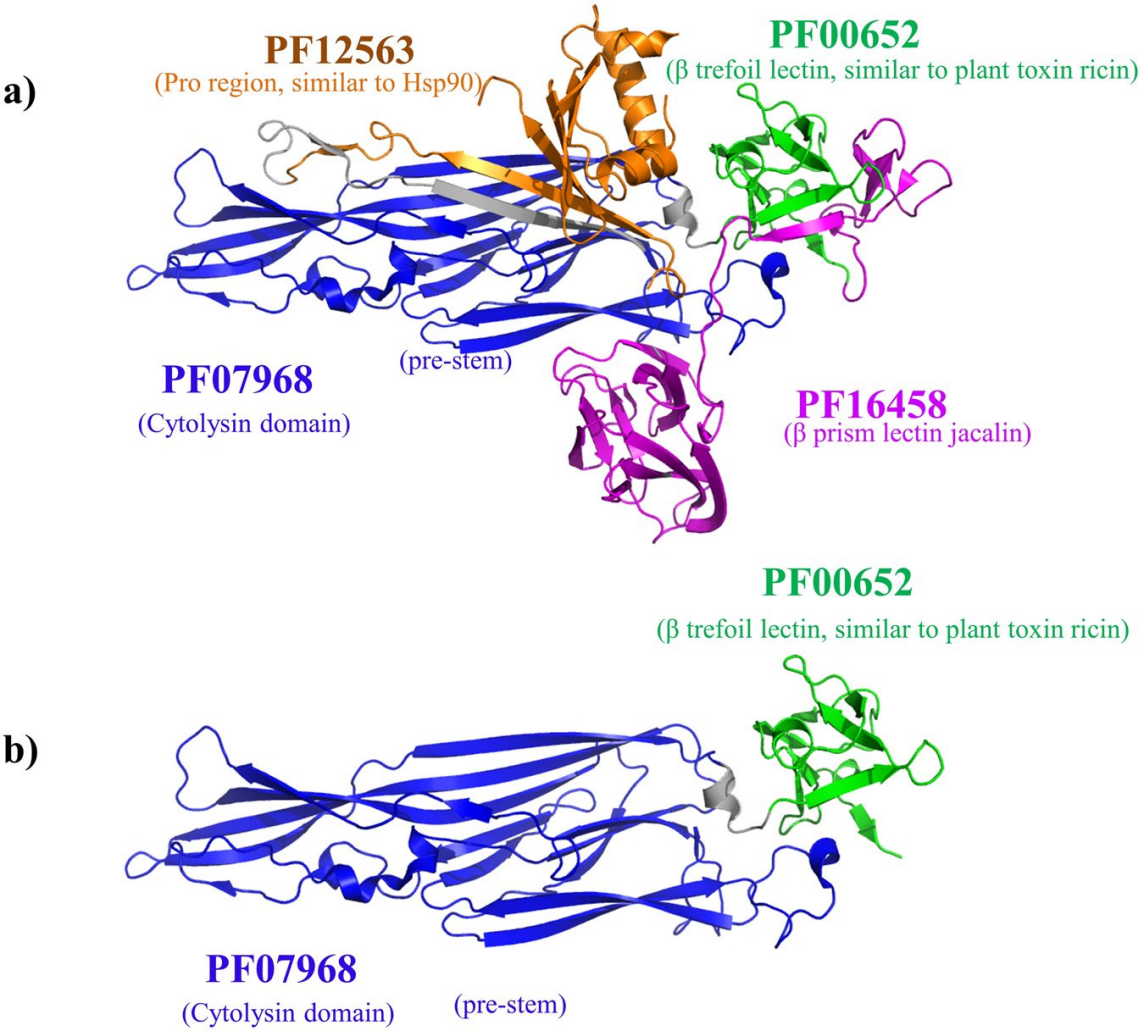
Domain structures of the hemolysins from *Vibrio cholera* (**a**) and *Vibrio vulnificus* (**b**). The membrane-active form of both is a heptameric, pore-forming structure.

The PFAM analysis clarifies the functional diversity of the large, multidomain proteins in the selected list of toxins. Many of the most studied bacterial toxins have several domains, which individually may serve regulatory or enzymatic functions not related to the pathogenic or cytotoxic effects of the whole protein^31^. For example, the hemolysin, HlyA of *Vibrio cholera* consists of four distinct domains, which all play a role in pore formation (Fig. 5A). The Pro region (PF12563) is cleaved to activate the toxin^32,33^, while the beta trefoil (PF00652), the cytolysin (PF07968) and beta prism lectin jacalin (PF16458) domains form a heptameric pore^34^. The homologous hemolysin toxin in *Vibrio vulnificus* (VVH) does not contain the beta prism lectin domain (PF16458) (Fig. 5B)^35^, while the chaperone-like Pro-domain is expressed as a separate gene product. The structural and sequence similarity between these toxins suggests that the VVH also forms a heptameric pore. Thus, even a high sequence similarity of a protein to the Pro or beta prism lectin region alone does not imply that the protein has a potential toxic effect, if the cytolysin and the beta trefoil lectins are absent. This example illustrates how a domain based approach can help in assessing functional similarity of a protein to known toxins.

### Sequence signatures distinguish toxic from non-toxic proteins within a protein family

**Figure 6:**
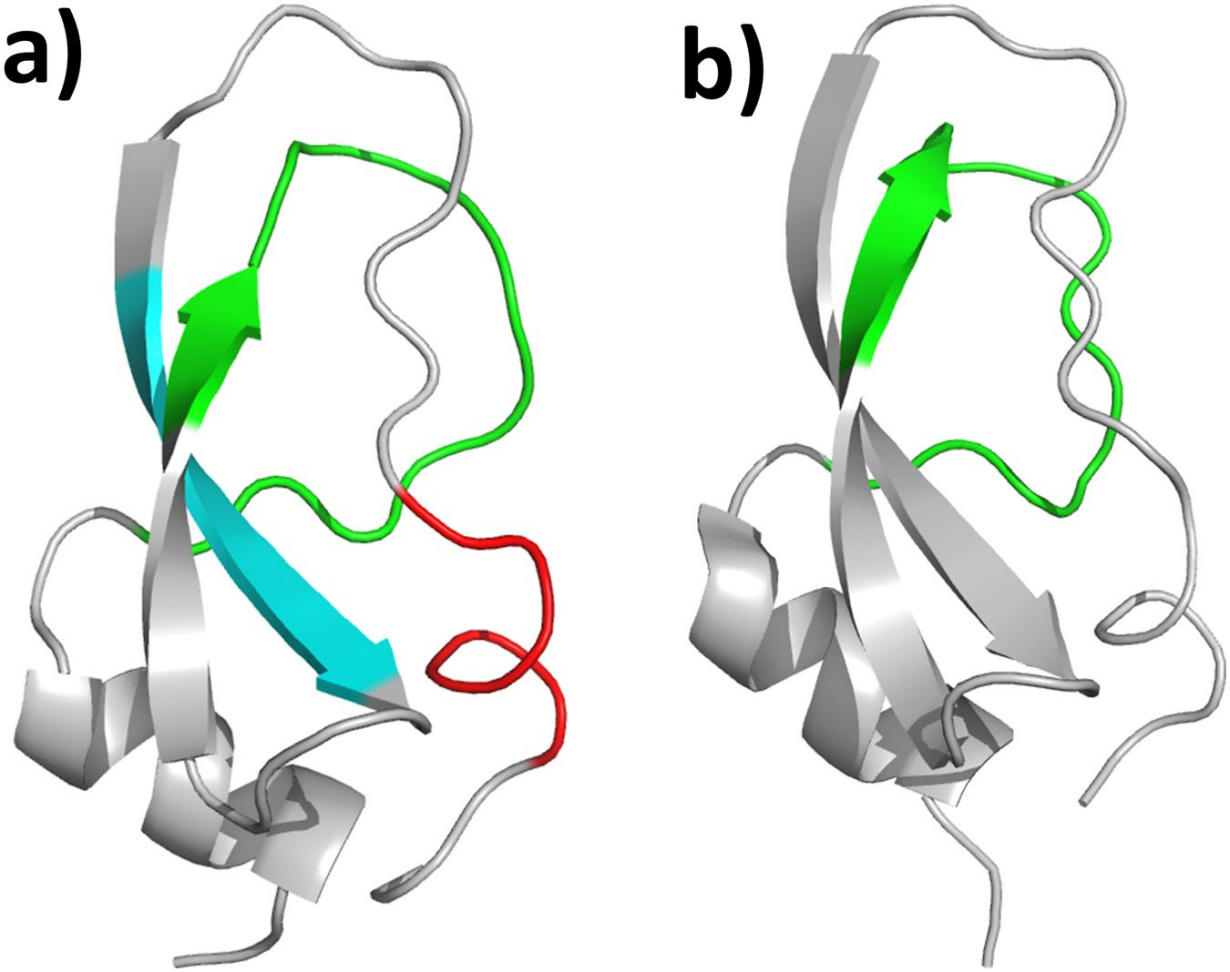
Dendrotoxin (**a**) and BPTI (**b**) group to the same Kunitz inhibitor PFAM domain and share the same 3D fold. Sequence motifs were generated in an alignment of 10 dendrotoxins (motif 1 in red, 2 in cyan and 3 in green). Only motif 3 had a significant score in trypsin inhibitors.

The diverse Kunitz-type protease inhibitor family includes (non-toxic) aprotinin or bovine pancreatic trypsin inhibitor (BPTI), domains of the Alzheimer’s amyloid precursor protein (APP) and tissue factor pathway inhibitor (TFPI). The same PFAM also contains toxic proteins from snake venoms^36^, including dendrotoxins from the venoms of mamba (*Dendroaspis*) snakes^37^. These dendrotoxins are highly homologous to BPTI, share the small (~6 kDa) prototype structure^38–40^ (Fig. 6), but function by blocking subtypes of voltage-dependent potassium channels of the Kv1 subfamily in neurons^41^. A BLAST sequence search in the NCBI sequence database with BPTI identified several dendrotoxins, with highly significant E-values of 10^−10^ to 10^−12^. However, dendrotoxins have little or no protease inhibitor activity and BPTI does not block potassium channels^37^. Therefore, E-values obtained from a BLAST search alone are not sufficient to distinguish toxic from non-toxic proteins in this family.

A functional motif analysis with PCPMer^18^ successfully distinguished the two protein groups. An alignment of experimentally verified potassium channel blocking dendrotoxins yielded 3 PCP- motifs (1: 25 KYCKLP 30, 2: 41 PSFYYK 46, 3: 55 FDYSGCGGNANRF 67). The three motifs were then searched with PCPMer in eight trypsin inhibitor sequences, including that of BPTI. For motif 1, the average and standard deviations of the score values are 0.89 ± 0.3 for the toxic dendrotoxins *versus* 0.68 ± 0.8 for non-toxic members (P value 1.0*10^−4^), and for M2 0.87 ± 0.07 versus 0.56 ± 0.01 (P-value 3*10^−7^). The values for motif 3 are not significantly different (P value 0.03) (Table S5). Thus, motifs 1 and 2 are uniquely found in toxic members, whereas motif 3 is found in toxic and non-toxic members. As the mapping on the structure (Fig. 6) shows, motif 1 overlaps with the amino acid residues that have been shown by site-directed mutagenesis to be critical for inhibiting potassium channels^41^. Thus, the motif analysis coincides well with experimental results for areas responsible for the different activities.

## Discussion

The objective of our research was to establish bioinformatics tools that can be used in the first tier of assessing the safety of proteins. The study, building on current industry practices to compare protein sequences to internal databases of known protein toxins, provides a validation of these approaches with quantitative data on the distribution of toxins in the protein landscape. We show here that known toxins belong to a restricted number of functional groups, as indicated by both a cluster analysis and specific annotation according to the PFAM classification. In addition, we demonstrate that motif recognition tools can distinguish the toxicity hazard of protein members within the same protein family. A detailed comparison will then allow the reviewer to determine the potential and possible mechanism for protein toxicity based on sequence or domain similarities with known toxic proteins. Such screening may eliminate unnecessary *in vivo* toxicity testing of a protein with valuable traits.

Our work demonstrates that the potential toxin sequences can be clustered into approximately 400 distinct groups, based on either sequence identity alone (Fig. 2a) or sequence features that link them to known functional protein families in PFAM (Fig. 4). As the list of potential toxins was independently established by four different research groups with keyword searches and inclusion of specific known toxins, we believe that the selected list of toxins covers most currently identified toxins. Thus, our finding that the number of protein toxin domains represents only a small fraction of all those known for proteins opens an opportunity to focus the safety assessment on a well-defined list of protein domains and their related functional activities.

As we have shown, sequence identity and even structural similarity cannot be used as sole criteria for defining a protein as a toxin. Some toxins might require specific protein binding sites to guide the toxin to its target, e.g. an ion channel in the case of dendrotoxins. In other cases several proteins act together in a complex. In the anthrax toxin, the two enzymatic proteins, lethal factor (LF), a metalloprotease, and edema factor (EF), an adenylyl cyclase, require the pore forming protective antigen to enter cells and exert a toxic effect^42–45^. For those cases motif analysis tools can be helpful to identify the necessary catalytic activities and protein binding sites for toxicity.

Further computational analysis is required to establish a standardized database containing validated motifs of toxins, *i.e.* proteins that when administered to vertebrates have an adverse effect. One current bottleneck to assembling a database is the non-standard nomenclature used by biologists for proteins that are very similar to one another, as noted above for Shiga toxin 2 A/verocytotoxin. On the other hand, proteins with the same name and similar functions, such as hemolysins, can have quite different domain structures and/or sequences depending on the organism (Fig. 5). Although efforts are underway to standardize the nomenclature of toxins from specific organisms such as spiders^6^, scorpions^46^, centipedes^47^ and snakes^48^, a unifying standard nomenclature for all toxins reflecting their structural and functional similarities is currently not available. The sequence clustering we have achieved here will help to clarify such nomenclature issues by assigning most of the toxins to PFAM domains, and hierarchical clustering of the toxin sequences at the three identity levels of 95%, 65% and 35%.

The most highly studied toxins produced by bacteria, as Table S3 indicates, have many different pathogenic mechanisms/modes of action, including ribonucleases (YoeB), vacuolating cytotoxins, hemolysins, cytolysins^49^, proteases, phospholipases^50^, leukotrienes, neurotoxins or pore formation^51,52^. The snake venom toxins and the conotoxins also present many different activities that can inhibit the growth of plant, insect or mammalian cells or block neural cell receptors. Another example of intrinsic diversity within proteins with similar functions are the vacuolating cytotoxins of *Helicobacter*. These form at least 8 distinct clusters even at 35% identity, emphasizing that the annotated function covers several distinct families with similar annotations. This is also seen for the conotoxins, which despite their short sequences cluster into different functional families.

Our analysis is designed to be a first screening stage, on which to base more detailed computational and experimental investigations. The final use for the protein depends on the risk versus benefits analysis and is outside the scope of this article. For example, even the most virulent proteins can have potentially valuable traits, depending on the administered dose and the route of exposure^53^. Ricin and Botulinum toxins are highly toxic at very low doses^54^, yet local injections of the later have proven useful for many therapeutic applications as well as the more widely publicized and profit-generating cosmetic ones. Ricin’s effects vary greatly depending on whether it is injected or consumed orally. Inactivated pertussis toxin is both a vaccine and a potential adjuvant^55^. Similarly, anthrax toxins and derivatives may have use as antitumor agents^56,57^. Rendering a protein toxic may also require posttranslational processing^58^, specific cofactors for activity^59^, or contact with specific receptors on target cells^60^ to exert pathogenic effects.

In conclusion, our current data suggest that there are only a few hundred sequentially and functionally distinct toxin clusters. This implies that most likely, the majority of proteins selected will not share those biochemical functions and can be considered as safe. For those that do bear some similarity to known toxins, we have summarized the basic functions of the largest toxin clusters and present a complete list of all PFAM domains for those toxins. This, coupled with motif recognition tools, provide the first stages of a possible approach to address functional similarities for novel protein products.

## Materials and Methods

### Selection of toxins

The basis for our work is a collection of potentially toxic proteins that were assembled in internal databases of four biotechnology companies: DuPont Pioneer, Bayer Crop Science, Monsanto and BASF. The sequences were selected using keyword searches (e.g., ‘toxic’, ‘toxin’) in the GenBank database, and specific toxins as published in the toxin list 40 CFR 725.421 of the EPA or from the UniProt animal toxin database^11^ were added. As keyword searches are not highly specific, proteins from safe organisms, short sequences with 100% sequence identity to longer entries, and those known to be non-toxic were removed (Fig. 1). The specific lists of keyword searches and the filtering processes were done independently by the research teams in the four companies. Finally, the combined database contained 10,389 sequences whose gi entries occurred in at least two of the databases. This selection criterion minimized the number of non-toxic entries in the database and at the same time gave comprehensive coverage of sequences with toxin annotations.

### Cluster analysis and functional classification

Cluster analysis of the toxin sequences was done with BLASTCLUST, a standalone software package distributed from NCBI. BLASTCLUST automatically clusters protein sequences based on pairwise alignments generated by the BLAST algorithm using the sequence identity and coverage of the alignment as a criterion to determine if the two sequences are neighbors. Clusters are generated by the single-linkage method, which includes a sequence in a cluster if the sequence is a neighbor to at least one sequence in the cluster. Classification of protein domains occurring in the toxins was based on the PFAM classification (version 29.0, Dec 2015 release). PFAM^17^ is a manually curated database of protein domains that contained 16295 entries. The identification of a domain for all toxin sequences in the database was determined by the HMMER^30^ software.

### Network analysis

Each sequence entry in the database received a unique identifier (cluster ID) for the membership in a cluster of a certain sequence identity level. For example, we denoted the clusters at the 95% level as Axxxx, where xxxx is the rank of the cluster among the 95% level clusters sorted according to the number of members. Thus A5 is the fifth largest cluster among the 95% level clusters. The IDs for the 65% and 35% level clusters were Dxxxx and Gxxxx respectively. A computer program in Perl was written to collect for each member in a cluster of the 35% level the memberships in the 65% and 95% clusters. The result was then represented and analyzed using Cytoscape^26^.

### Motif analysis

Homologous proteins with similar function usually share similar sequence regions, although the overall sequence identity can be as low as 20–30%. These critical regions, also known as motifs, important for the biological function and similar fold, are in most cases highly conserved^61–64^. In this study, we used PCPMer^18,20,65^ to generate motifs of a toxin family and then used these motifs to search for similar sequence regions  in other proteins. PCPMer identifies functionally important areas  based on conservation of physical-chemical properties (PCPs) of amino acids in a multiple sequence alignment of proteins. The criteria for conservation are derived from the distributions of the PCP descriptors in each column of the alignment as compared to a background distributions, derived from a statistical study of non-redundant proteins from the Swiss-Prot database^66^ as a random sample. If the distributions of the five PCP descriptors are significantly different from the background distribution as measured by the relative entropy (or Kullback-Leibler divergence)^67^ for any of the five descriptors E1 to E5, that position is considered as conserved. The functional motifs are defined as continuous stretches of conserved residues with relative entropy values higher than an empirical or user specified threshold. The motifs are typically 5–15 amino acids in length, where the minimum length and inclusion of gaps can be specified by the user. The PCPMer approach has been successfully used to characterize functionally important sites in endonucleases, the cytochrome P450 protein, metal-binding proteins, the Ig domains of the muscle protein titin and several allergenic proteins^18,42,68–72^.

### Data Availability

The complete list of PFAM domains containing toxic proteins (Table S4) can be downloaded from our website http://curie.utmb.edu/SciRep/Negi_et_Table_S4.xlsx. All other data generated or analyzed during this study are included in this published article and its Supplementary Information files.

## Electronic supplementary material

Supplementary Material
