# Supplementary material for: Functional classification of protein toxins as a basis for bioinformatic screening
Source: Sci Rep. 2017 Oct 24;7:13940. doi: 10.1038/s41598-017-13957-1 (PMC5655178; doi:10.1038/s41598-017-13957-1)
Supplement: Supplementary file 1 — Supplementary Material [file 41598_2017_13957_MOESM1_ESM.pdf]

Supplementary Material

Functional classification of protein toxins as a basis for bioinformatic screening

Surendra S. Negi[1], Catherine H. Schein[1,2], Gregory S. Ladics[3], Henry Mirsky[4], Peter Chang[4], Jean-Baptiste Rascle[5], John Kough[6], Lieven Sterck [7], Sabitha Papineni[8], Joseph M. Jez[9], Lucilia Pereira Mouriès[10], Werner Braun[1]*

[1]Sealy Center for Structural Biology and Molecular Biophysics, Department of Biochemistry and Molecular Biology, University of Texas, Medical Branch, Galveston, TX 77555-0304, USA.
[2]Foundation for Applied Molecular Evolution, Inc., Alachua, FL 32615-9495, USA.
[3]DuPont Haskell Laboratory, 1090 Elkton Road, Newark, DE 19711, USA.
[4]Pioneer Hi-Bred, DuPont Agricultural Biotechnology, 200 Powder Mill Road, Wilmington, DE 19880, USA.
[5]Bayer SAS, 355 rue Dostoïevski, CS 90153 Valbonne, 06906 Sophia Antipolis, France.
[6]Office of Pesticide Programs, Microbial Pesticides Branch, US Environmental Protection Agency, Washington, DC, USA.
[7]Department of Plant Systems Biology, Department of Plant Biotechnology and Bioinformatics, Ghent University, B–9052 Ghent, Belgium.
[8]Dow AgroSciences LLC, 9330 Zionsville Road, Indianapolis, IN 46268, USA.
[9]Department of Biology, Washington University in St. Louis, One Brookings Drive, CB 1137, St. Louis, MO, USA.
[10]ILSI Health and Environmental Sciences Institute (HESI), 1156 Fifteenth St., NW, Washington, DC 20005, USA.

*: Corresponding author W. Braun. Email: webraun@utmb.edu, Telephone (409) 747-6810

Table S1 Top 100 clusters at the 95% sequence identity level:

Cluster 1 cytotoxin associated protein A
Cluster 2 Shiga toxin 2 A / verocytotoxin 2 A
Cluster 3 vacuolating cytotoxin, partial
Cluster 4 vacuolating cytotoxin
Cluster 5 Hainantoxin-II-10.2
Cluster 6 Hainantoxin-XVI-10
Cluster 7 Shiga toxin 2 B / verocytoxin 2 B
Cluster 8 vacuolating cytotoxin A
Cluster 9 Enterotoxin / mitogenic exotoxin
Cluster 10 vacuolating cytotoxin
Cluster 11 U1-lycotoxin-Ls1a
Cluster 12 alpha toxin [Clostridium perfringens]
Cluster 13 enterotoxin
Cluster 14 vacuolatin cytotoxin
Cluster 15 U3-lycotoxin-Ls1a
Cluster 16 Hainantoxin-VIII
Cluster 17 Hainantoxin-III / Mu-theraphotoxin-Hhn2a
Cluster 18 vacuolating cytotoxin
Cluster 19 botulinum neurotoxin type B [Clostridium botulinum]
Cluster 20 Phospholipase D LapSicTox-alpha / Dermonecrotic toxin / Sphingomyelin phosphodiesterase D
Cluster 21 enterotoxin
Cluster 22 vacuolating cytotoxin
Cluster 23 enterotoxin C
Cluster 24 RTX toxin RtxA
Cluster 25 cholix toxin
Cluster 26 Shiga toxin subunit A
Cluster 27 cardiotoxin / cytotoxin
Cluster 28 Vacuolating cytotoxin
Cluster 29 enterotoxin
Cluster 30 Hainantoxin-XIII
Cluster 31 neurotoxin 1
Cluster 32 alpha-toxin
Cluster 33 Phospholipase D / Dermonecrotic toxin / Sphingomyelin phosphodiesterase D
Cluster 34 phospholipase C alpha toxin
Cluster 35 vacuolating cytotoxin
Cluster 36 Omega-conotoxin
Cluster 37 Conotoxin Ebx.x
Cluster 38 vacuolating cytotoxin (VacA)
Cluster 39 Enterotoxin
Cluster 40 cystine knot toxin [Dolomedes mizhoanus]
Cluster 41 enterotoxin
Cluster 42 cytotoxin associated protein A
Cluster 43 U8-lycotoxin-Ls1a / Toxin-like structure LSTX-Hx
Cluster 44 four-loop conotoxin LVVIx

Cluster 45 hemolytic/non-hemolytic enterotoxin
Cluster 46 Enterotoxin
Cluster 47 beta-bungarotoxin chain Ax / Phospholipase A2
Cluster 48 vacuolating cytotoxin
Cluster 49 O-superfamily conotoxin
Cluster 50 cytolytic enterotoxin
Cluster 51 bontoxilysin A / botulinum neurotoxin
Cluster 52 Phospholipase D LhSicTox-alphaIA2ai / Dermonecrotic toxin/ Sphingomyelin phosphodiesterase D
Cluster 53 vacuolating cytotoxin
Cluster 54 alpha-latrotoxin
Cluster 55 cholera enterotoxin B subunit
Cluster 56 U1-lycotoxin-Ls1aa / Toxin-like structure LSTX-Axx
Cluster 57 cholix toxin / Exotoxin A
Cluster 58 exotoxin A / NAD(+)--diphthamide ADP-ribosyltransferase
Cluster 59 pyrogenic exotoxin B / Strepotococcal cysteine protease (Streptopain)
Cluster 60 vacuolating cytotoxin A
Cluster 61 vacuolating cytotoxin protein A
Cluster 62 nonhemolytic enterotoxin A
Cluster 63 cytolethal distending toxin B subunit
Cluster 64 alpha toxin
Cluster 65 M-zodatoxin-Lt8a / Cytoinsectotoxin-1x
Cluster 66 Kunitz-type serine protease inhibitor hainantoxin-XI-x
Cluster 67 vacuolating cytotoxin A
Cluster 68 leukotoxin
Cluster 69 diphtheria toxin / NAD(+)--diphthamide ADP-ribosyltransferase
Cluster 70 pertussis toxin subunit 1
Cluster 71 cytolethal distending toxin A
Cluster 72 vacuolating cytotoxin
Cluster 73 U15-lycotoxin-Ls1a / Toxin-like structure LSTX-Nx;
Cluster 74 alpha-bungarotoxin
Cluster 75 cytotoxin associated protein A
Cluster 76 beta 2 toxin
Cluster 77 vacuolating cytotoxin
Cluster 78 Shiga toxin 1 B subunit
Cluster 79 Hainantoxin-IX-x.x
Cluster 80 O-superfamily conotoxin
Cluster 81 cyotoxin x
Cluster 82 alpha-conotoxin
Cluster 83 cytotoxin K
Cluster 84 U13-lycotoxin-Ls1x / Toxin-like structure LSTX-Lx
Cluster 85 vacuolating cytotoxin
Cluster 86 U6-lycotoxin-Ls1x / Toxin-like structure LSTX-Fx
Cluster 87 Omega-conotoxin / four-loop conotoxin
Cluster 88 enterotoxin B
Cluster 89 nonhemolytic enterotoxin B

Cluster 90 exotoxin G variant x / pyrogenic exotoxin G
Cluster 91 vacuolating cytotoxin
Cluster 92 ammodytoxin / Basic phospholipase A2 ammodytoxin
Cluster 93 leukotoxi
Cluster 94 toxin Css39.8 / Beta-toxin / sodium-channel modifier toxin precursor CsEv2x
Cluster 95 Hainantoxin-XIV-x
Cluster 96 cobrotoxin homolog / Short neurotoxin / siamenotoxin
Cluster 97 cardiotoxin 1
Cluster 98 U8-lycotoxin-Ls1x / Toxin-like structure LSTX-Hx
Cluster 99 I-superfamily conotoxin
Cluster 100 M-superfamily conotoxin

Table S2. Complete list of headers in the toxins for each of the top 100 35% sequence identity clusters

1. alphaO-conotoxin GeXIVAWT,conotoxin,conotoxin scaffold,four-loop conotoxin A,mu-O conotoxin MrVIB ,O superfamily conotoxin ,O1-conotoxin peptide precursor ,omega-conotoxin GVIA precursor,Delta-conotoxin,Omega-conotoxin ,Kappa-conotoxin,
2. cra-3 toxin,alpha naeorotoxin(1-3), beta toxin ,beta-buthitoxin, Cex(1-10) neurotoxin precursor, CsE M1=toxin,depressant scorpion toxin KIM2 precursor gamma-b=toxin gamma homolog,III-8b=toxin III-8 homolog,iota-buthitoxin-Hj1a,IV-5b=toxin,long-chain sodium channel specific toxin 8,makatoxin II,Na+ channel-specific toxin 2,Na+-channel blocking toxin,Na+-channel modifying toxin precursor,neurotoxin KIT, neurotoxin(all), Alpha-insect toxin, Alpha-mammal toxin, Alpha-toxin, Ardiscretin, Anti-neuroexcitation peptide 3, Beta-insect depressant toxin, Beta-insect excitatory toxin, Beta-mammal toxin, Beta-toxin, Bukatoxin, Depressant insect toxin, Insect toxin, Lipolysis-activating peptide, Makatoxin, Neurotoxin, Toxin Aah, Toxin BmK, scorpion toxin, sodium channel alpha-toxin Acra4 precursor, sodium channel neurotoxin alpha type, sodium channel toxin, sodium toxin peptide, sodium-channel modifier toxin , U2-buthitoxin-H1a/Hj1a/, venom sodium channel toxin(1-9)
3. alpha neurotoxin, alpha-bungarotoxin deletion, cardiotoxin, erabutoxin, gamma-bungarotoxin, kappa 1a bungaratoxin, long chain alpha-neurotoxins, neurotoxin, non-conventional three finger toxin isoform, Alpha-bungarotoxin isoform, Alpha-elapitoxin, Beta-cardiotoxin, Cardiotoxin homolog , Cytotoxin , Denmotoxin, Haditoxin, Irditoxin subunit A, Long neurotoxin, Muscarinic toxin , Three finger toxin, Tryptophan-containing weak neurotoxin, scutelatoxin, short chain alpha neurotoxin, short-chain three finger toxin isoform, siamenotoxin I precursor, three finger toxin,
4. cytotoxin associated protein,
5. addiction module toxin (Txe/YoeB), addiction module toxin,plasmid encoded toxin ,RelE family toxin-antitoxin system toxin component, toxin RelK, Toxin YoeB, toxin-antitoxin system, toxin component, Txe/YoeB family, txe/YoeB family addiction module toxin
6. enterotoxin, exotoxin, extracellular enterotoxin L, mitogenic exotoxin, staphylococcal enterotoxin , Superantigen enterotoxin SEK/SEL
7. alpha-bungarotoxin, alpha-delta-bungarotoxin,cardiotoxin, kappa-toxin,long chain alpha-neurotoxins, muscarinic toxin, Acetylcholinesterase toxin C; Adrenergic toxin, Alpha-elapitoxin, Alpha-bungarotoxin, Cobrotoxin, Cytotoxin, Fasciculin, Frontoxin , Long neurotoxin, Muscarinic toxin , Oxiana weak toxin, Rho-elapitoxin-Da1b, Pseudonajatoxin b, Pelamitoxin a, Short neurotoxin
8. dermonecrotic toxin isoform, Phospholipase D LafSicTox,
9. A superfamily conotoxin, A-conotoxin peptide precursor, alpha conotoxin A-superfamily , alpha conotoxin, conotoxin, Alpha-conotoxin, Kappa-conotoxin, superfamily conotoxin
10. cystine knot toxin, Hanatoxin, huwentoxin-I, Omega-GrammotoxinSIA, Kappa-theraphotoxin, Mu-theraphotoxin, U17-theraphotoxin, venom toxin peptide
11. addiction module relE-like toxin, Addiction module toxin, addiction module toxin, RelE/StbE, Cytotoxin, RelE protein, Plasmid stabilization system addiction module toxin, RelE antibacterial toxin protein , RelE toxin, RelE-like addiction module toxin , replicon stabilization toxin, StbE replicon stabilization toxin , toxin-antitoxin system

12. conotoxin, T superfamily conotoxin, T-1-conotoxin pu5a precursor , tau conotoxin
13. Conomarphin conotoxin precursor analog, conotoxin, kappaM conotoxin, M conotoxin, M superfamily conotoxin, mu conotoxin SIIIA , M-superfamily conotoxin,
14. VacA cytoxin,vacuolating cytotoxin
15. addiction module toxin, addiction module toxin, RelE/StbE, toxin of the YafQ-DinJ toxin-antitoxin system, toxin-antitoxin system,YafQ toxin protein
16. Omega-ctenitoxin, U1-lycotoxin-Ls1, U3-lycotoxin-Ls1, U4-lycotoxin-Ls1, U5-lycotoxin-Ls1
17. vacuolating cytotoxin,
18. Shiga toxin subunit, Shiga-like toxin, variant Shiga toxin , verocytotoxin 2 subunit, verotoxin-2 variant subunit A
19. A1 chain of beta-bungarotoxin,ammodytin, ammodytoxin B/C, beta bungaratoxin A, beta-neurotoxin, bothropstoxin-Ia, myotoxic protein PLA2K49 , Acidic phospholipase A2 beta-bungarotoxin, Basic phospholipase A2, Neutral phospholipase A2 agkistrodotoxin, Phospholipase A2 crotoxin, scutoxin precursor, textilotoxin, venom gland myotoxin I
20. conotoxin, O-conotoxin , O-superfamily conotoxin, O2 superfamily conotoxin, XV conotoxin,
21. bontoxilysin A, botulinum neurotoxin, C/D mosaic neurotoxin, neurotoxin, tetanus toxin, type B cryptic neurotoxin,
22. enterotoxin, viral enterotoxin,
23. Addiction module toxin, Death on curing protein,, ParE-like toxin of addiction system, RelE/StbE family addiction module toxin, toxin-antitoxin system, YafQ toxin protein
24. vacuolar cytotoxin,acuolating cytotoxin,
25. vacuolating cytotoxin,
26. cytolethal distending toxin, type III cytolethal distending toxin protein CdtB
27. vacuolating cytotoxin,
28. vacuolating cytotoxin
29. Exotoxin, Staphylococcal exotoxin, superantigen-like protein,
30. cystine knot toxin, huwentoxin, LTx2 toxin, LTx3 toxin, toxin ba3 precursor
31. Shiga toxin, Shiga-like toxin, shigatoxin, variant Shiga toxin type 2 B, verocytotoxin 2 subunit B
32. LTx4 toxin, Hainantoxin, Mu-hexatoxin-Mg1a, U26-theraphotoxin, U3-theraphotoxin
33. U10-lycotoxin-Ls1, U11-lycotoxin-Ls1, U6-lycotoxin-Ls1, U7-lycotoxin-Ls1, U8, U9 lycotoxin-Ls1
34. conotoxin, I-superfamily conotoxin, 2-conotoxin peptide precursor, Kappa-conotoxin-like,
35. cytotoxin, exotoxin paxA, hemolysin toxin protein, Leukotoxin, RTX A toxin , RTX-toxin
36. enterotoxin, haemolytic enterotoxin, non hemolytic enterotoxin , non-expressed Enterotoxin
37. cytolytic enterotoxin,
38. alpha toxin,

39. beta toxin , Beta/delta-agatoxin, U2-agatoxin-Ao1/U3/U5
40. cystine knot toxin, lycotoxin(u14,U15,U7)
41. Cangitoxin, Major neurotoxin BcIII, Neurotoxin, Toxin AETX/APE/CgNa/Hk/PCR/Rc
42. bromosleeper conotoxin O3-superfamily protein, conotoxin, O3 superfamily conotoxin
43. anntoxin, anntoxin S(1-5), beta bungaratoxin B, bungarotoxin, dendrotoxin K,Kunitoxin-Phi1,Kunitz-type neurotoxin, potassium channel peptide toxin
44. BeKm-1 toxin precursor, KT neurotoxin precursor, neurotoxin KT, Potassium channel toxin alpha-KTx, Neurotoxin alpha-KTx, toxin AaTX, TX1 toxin precursor , U8-buthitoxin-Hj3a
45. neurotoxin, peptide toxin , sodium channel neurotoxin, sodium channel peptide toxin , sodium channel toxin
46. cholix toxin,
47. vacuolating cytotoxin,
48. cytolethal distending toxin
49. Hok/gef cell toxic protein, Qin prophage; small toxic membrane peptide
50. cytolethal distending toxin A,
51. cytolethal distending toxin B,
52. zeta toxin, anititoxin/toxin system zeta toxin, bacterial zeta toxin, PezT Zeta toxin ,
53. alpha-latrotoxin,
54. alphaD-mus conotoxin precursor, conotoxin, Alpha-conotoxin
55. K(+)-channel-blocking toxin 1, margatoxin,noxiustoxin 2, Potassium channel toxin alpha-KTx
56. Aerolysin/hemolysin/leukocidin toxin
57. conotoxin, J-conotoxin peptide precursor Fla-, J-superfamily conotoxin, Alpha/kappa-conotoxin
58. toxin outer membrane protein, vacuolating cytotoxin (VacA)
59. cholix toxin,
60. exfoliative toxin/A, K11041 exfoliative toxin A/B
61. Enterotoxin, exotoxin, pyrogenic exotoxin protein, streptococcal group G pyrogenic exotoxin G, treptococcal pyrogenic exotoxin J
62. ergtoxin precursor,Potassium channel toxin gamma-KTx
63. Exotoxin,
64. vacuolating cytotoxin,
65. Kunitz-type serine protease inhibitor hainantoxin-XI
66. cholera enterotoxin,enterotoxin subunit A, heal labile enterotoxin subunit, heat-labile enterotoxin
67. enterotoxin / cell-wall binding protein, enterotoxin
68. enterotoxin, haemolytic enterotoxin , non hemolytic enterotoxin,
69. RTX toxin RtxA,
70. alpha conotoxin, superfamily conotoxin,
71. cholera enterotoxin B, cholera toxin, enterotoxin subunit B, heat-labile enterotoxin B,

72. CcdB toxin protein, CcdB-like toxin protein, cytotoxic protein, post-segregation toxin CcdB, Toxin addiction system:
73. M vacuolating cytotoxin, vacuolating cytotoxin precursor,
74. addiction module toxin, HigB addiction module toxin, HigB toxin, Plasmid maintenance system killer protein; Toxin higB-1, toxin, RelE family , toxin-antitoxin system,
75. addiction module toxin, RelE/StbE family
76. I-superfamily conotoxin,Iota-conotoxin,
77. toxin Fic, toxin-antitoxin system,
78. phospholipase C alpha toxin,
79. V-conotoxin peptide precursor, V1 superfamily conotoxin 15.1
80. hrtoxin-a subunit, ijtoxin-a subunit, neoverrucotoxin a-subunit, patoxin-a subunit, pvtoxin-b, Stonustoxin subunit beta;, Verrucotoxin subunit beta,
81. Enterotoxin,
82. exfoliative toxin
83. conotoxin, I1 superfamily conotoxin, iota conotoxin I1-superfamily , Conotoxin
84. cytolethal distending toxin A
85. beta 2 toxin,cpb2 toxin,
86. vacuolating cytotoxin,
87. cytotoxin A,vacuolating cytotoxin precursor,vacuolating toxin VacA,
88. Cytotoxin / haemolysin homologue TlyA, Cytotoxin/haemolysin homologue
89. cytolethal distending toxin A,
90. vacuolating cytotoxin precursor
91. M-zodatoxin-Lt
92. cystine knot toxin, Kappa-ctenitoxin-Pn1a; Omega-ctenitoxin-Pn1a;,lycotoxin-Ls1(U9,U16,U17,U18,U6)
93. saxitoxin,
94. alpha-conotoxin,
95. alpha-latrocrustotoxin precursor, alpha-latrotoxin,
96. leukotoxin A
97. equinatoxin II precursor, fragaceatoxin C, gigantoxin-4, hemolytic toxin, urticinatoxin
98. Lectoxin-Enh(3-7), Lectoxin-Lei(1-3), lectoxin-Vind1, Lectoxin-Phi1-2, lectoxin-Oapo1,
99. cystine knot toxin, U13-lycotoxin-Ls1a;
100. cystine knot toxin, Delta-ctenitoxin-Pn1a, GAMMA-ctenitoxin-Pn1a;U(1,12) lycotoxin-Ls1

Table S3: The most populated bacterial toxin clusters at 65% and 35% identity are similar, indicating the larger clusters at the 35% identity level preserve the intrinsic granularity in the data. If there is no number in the middle column, the proteins were not included in the top 100 clusters at 65% identity.

**NCBI Annotation**	**Cluster # at 65% identity**	**Cluster # at 35% identity**
Helicobacter pylori toxins (vacuolating)	1,3,8,14,54.59,60,74,94,97	4,17,24,27,28,58,64,73,87,90
Yoe B/Txe families (Addiction module toxins)	5,44	5
Shiga toxins	13, 26, 68	18,31
RelE/Stb families stability protein (groups with Death on curing protein, Doc toxin [Thioalkalivibrio nitratireducens] Vibrio, YafQ, and others at 35%	33,75	11,15,23 74,75
Aeromonas hydrophila enterotoxin	34	37
S pyogenes exotoxin	35	61
C. perfringens alpha toxin (phospholipase)	38	38
Leukotrienes RTXII	47,69	35,
C, botulinus neurotoxin, bontoxilysin	50, 67	21
B. cereus and thurengiensis enterotoxin C haemolytic and non haemolytic enterotoxin	56,71,91	68
Staph. aureus enterotoxins (and other mitogenic exotoxin Z [*Streptococcus pyogenes*] at 35%)	58,65,99	6
cytolethal distending toxin B [Campylobacter coli] and other Campylobacter (and from Helicobacter, at 35%)	72,92	26. 48,50, 89
cholera enterotoxin B subunit [*Vibrio cholerae*]	73	71
Enterotoxin [*Bacillus anthracis* str. H9401] and other bacillus species, including cereus and thuriengiensis	87, 91	36, 67,81
Enterotoxin and exotoxin G [*Streptococcus pyogenes*]	88	
Beta 2 toxin, partial [Clostridium perfringens] (CPB toxin)	93	38 , 85 (g toxin)
Cytolethal distending toxin b, partial [*Helicobacter bilis*] and other Helicobacter	98	26
Exotoxin [*Staphylococcus aureus* subsp. aureus 71193]	100	29, 63
Leukotoxins and RTX I &III		35
Hok/gef cell toxic protein (plasmid) [Enterobacter asburiae LF7a] and small toxic membrane proteins from a variety of G- bacteria including coli and Klebsiella		50
cytolethal distending toxin B, partial [Escherichia coli]		51
bacterial zeta toxin protein [Streptococcus pyogenes] and others; toxin/antitoxin		52
Aerolysin/hemolysin/leukocidin toxin [*Shewanella baltica* BA175] and alpha toxin of *C. botulinus*		56
Cholix toxin, partial [Vibrio cholerae]		59
Exfoliative toxin [Streptococcus pyogenes M1 476]		60, 82
RTX toxin RtxA, partial (plasmid) [*Vibrio vulnificus*]		69
CcdB toxin protein (plasmid) [*Klebsiella pneumoniae*]		72
HigB [*Psychroflexus torquis* ATCC 700755]		74
Toxin-antitoxin system, toxin component, Fic family [*Fusobacterium nucleatum* subsp. vincentii 3_1_27]		77
C. perfringens B-toxin		85
Cytotoxin/hemolysin [*Mycobacterium leprae*]		88
Leukotoxin A, partial [*Pasteurellaceae* bacterium I10]		96
Viral enterotoxin Rotavirus	10	22

Table S4. Table S4 can be downloaded from our website at : http://curie.utmb.edu/SciRep/Negi_et_Table_S4.xlsx

Table S5. Score values of the three PCP motifs in dendrotoxins (top) and homologous trypsin inhibitors (bottom).

GI	Motif 1	Motif 2	Motif
125035	0.9	0.92	0.91
657341360	0.95	0.92	0.98
266399	0.9	0.92	0.91
385318	0.91	0.89	0.98
125047	0.91	0.89	0.98
169788687	0.83	0.89	0.98
1181912	0.89	0.89	0.98
1181914	0.87	0.89	0.98
125055	0.84	0.78	0.98
255642923	0.86	0.69	0.89

GI	Motif 1	Motif 2	Motif 3
115114	0.67	0.56	0.73
124075	0.63	0.56	0.73
115115	0.63	0.56	0.73
2497580	0.67	0.56	0.86
28202267	0.64	0.56	0.98
3915130	0.64	0.58	0.98
6647513	0.88	0.55	0.98
6435821	0.67	0.56	0.73
